# Supplementary material for: Human protein synthesis requires aminoacyl-tRNA pivoting during proofreading
Source: Nat Commun. 2025 Sep 2;16:8202. doi: 10.1038/s41467-025-63617-6 (PMC12405580; doi:10.1038/s41467-025-63617-6)
Supplement: Supplementary file 2 — Description of Additional Supplementary Files [file 41467_2025_63617_MOESM2_ESM.pdf]

Description of Additional Supplementary Files

File name: Supplementary Movie 1
Description: Accommodation of human aa-tRNA. eEF1A (purple) delivers aa-tRNA (yellow) to the ribosome, with 28S, 5S, and 5.8S rRNA (white); 18S rRNA (grey); peptidyl-tRNA (orange); ribosomal proteins (light blue); and 3' CCA ends (blue). The aa-tRNA begins in a bent position bound to eEF1A in the initial binding state. It then transitions from the bent state to the A/A site as it traverses the accommodation corridor. The first view is from the central protuberance, the second view is from the LSU, and the third view highlights the contributions of the accommodation corridor, including H69 (red), H71 (green), H90 (gray), H92 (pink), h19 (purple), and h44 (white).

File name: Supplementary Movie 2
Description: Accommodation of E. coli aa-tRNA. EF-Tu (purple) delivers aa-tRNA (yellow) to the ribosome, with 28S, 5S, and 5.8S rRNA (white); 18S rRNA (grey); peptidyl-tRNA (orange); ribosomal proteins (light blue); and 3' CCA ends (blue). The aa-tRNA begins in a bent position bound to EF-Tu in the initial binding state. It then transitions from the bent state to the A/A site as it traverses the accommodation corridor. The first view is from the central protuberance, the second view is from the LSU, and the third view highlights the contributions of the accommodation corridor, including H69 (red), H71 (green), H90 (gray), H92 (pink), h19 (purple), and h44 (white).
